# Supplementary material for: DNA binding redistributes activation domain ensemble and accessibility in pioneer factor Sox2
Source: Nat Commun. 2024 Feb 16;15:1445. doi: 10.1038/s41467-024-45847-2 (PMC10873366; doi:10.1038/s41467-024-45847-2)
Supplement: Supplementary file 1 — Supplementary information [file 41467_2024_45847_MOESM1_ESM.pdf]

Supplementary information

DNA binding redistributes activation domain ensemble and accessibility in pioneer factor Sox2

Sveinn Bjarnason[1#], Jordan A.P. McIvor[2#] Andreas Prestel[3], Kinga S. Demény[1], Jakob T. Bullerjahn[4], Birthe B. Kragelund[3], Davide Mercadante[2*], and Pétur O. Heidarsson[1*]

[1]Department of Biochemistry, Science Institute, University of Iceland, Sturlugata 7, 102 Reykjavík, Iceland.

[2]School of Chemical Science, University of Auckland, Auckland, New Zealand.

[3]REPIN and Structural Biology and NMR Laboratory, Department of Biology, University of Copenhagen, Ole Maaløes Vej 5, 2200 Copenhagen N, Denmark.

[4]Department of Theoretical Biophysics, Max Planck Institute of Biophysics, Max-von-Laue-Straße 3, 60438, Frankfurt am Main, Germany.

[#]These authors contributed equally: Sveinn Bjarnason, Jordan McIvor.

**Correspondence to davide.mercadante@auckland.ac.nz, pheidarsson@hi.is*

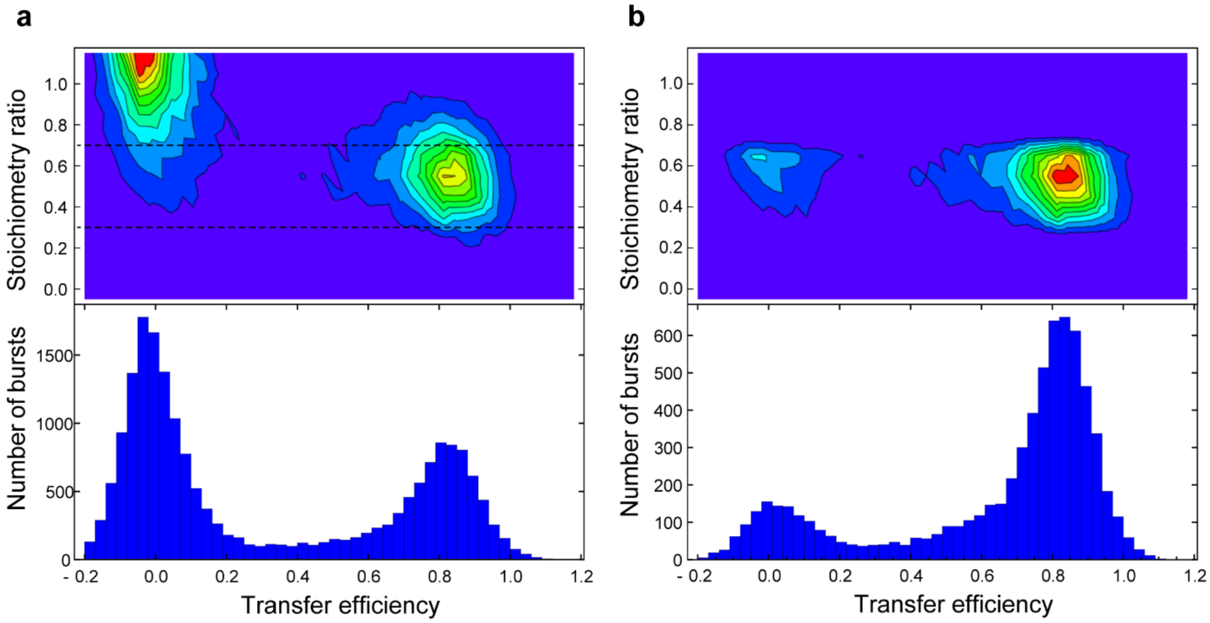

Supplementary Figure 1. Stoichiometry measurements using pulsed-interleaved excitation. a-b) 2D-histograms of stoichiometry ratio vs. transfer efficiency from intramolecular FRET of Sox2 labelled in positions 37 and 120, before **(a)** and after **(b)** PIE filtering. The dotted lines in panel a indicate the range of stoichiometry ratios used for filtering out donor-only bursts. In this analysis, a burst originating from a molecule that has an active donor and acceptor results in a stoichiometry ratio of 0.5. In some cases, an additional residual population at a transfer efficiency close to zero but with stoichiometry ~1 can remain even after filtering (panel b) due to a large signal from molecules lacking an active acceptor dye.

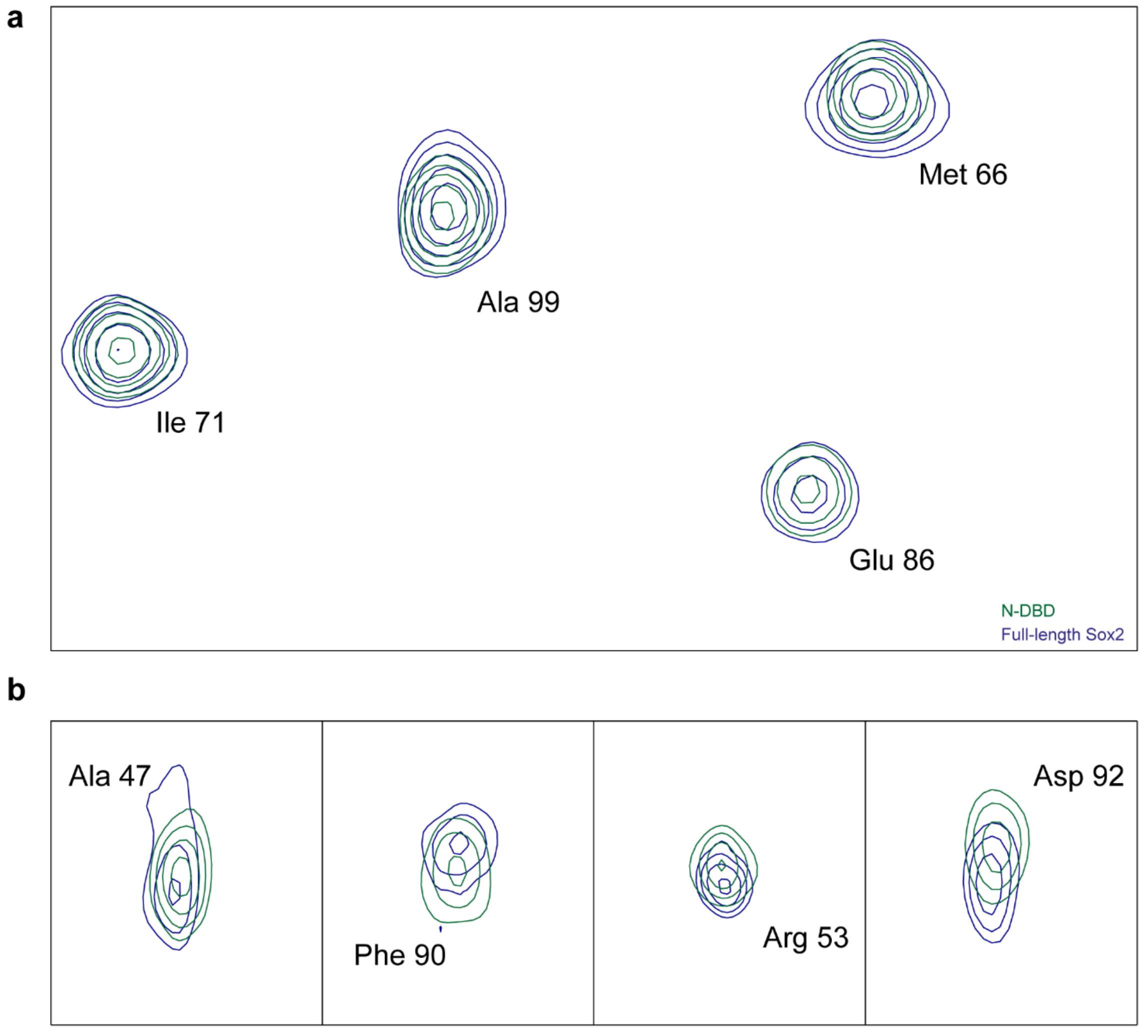

Supplementary Figure 2. Peak positions and intensities of Sox2 DBD. a-b) Examples of chemical shifts from $^{1}H^{15}N$ BTROSY[1] of full-length Sox2 (blue) overlapped with $^{1}H^{15}N$ BTROSY of the N-DBD (green). Most peaks from the DBD in the full-length protein generally overlap well with peaks from an isolated DBD (panel a) but many show small but specific chemical shift perturbations (panel b). This indicates that the DBD fold is generally unperturbed by the presence of the C-IDR. Both spectra were run with 640 scans at 15°C.

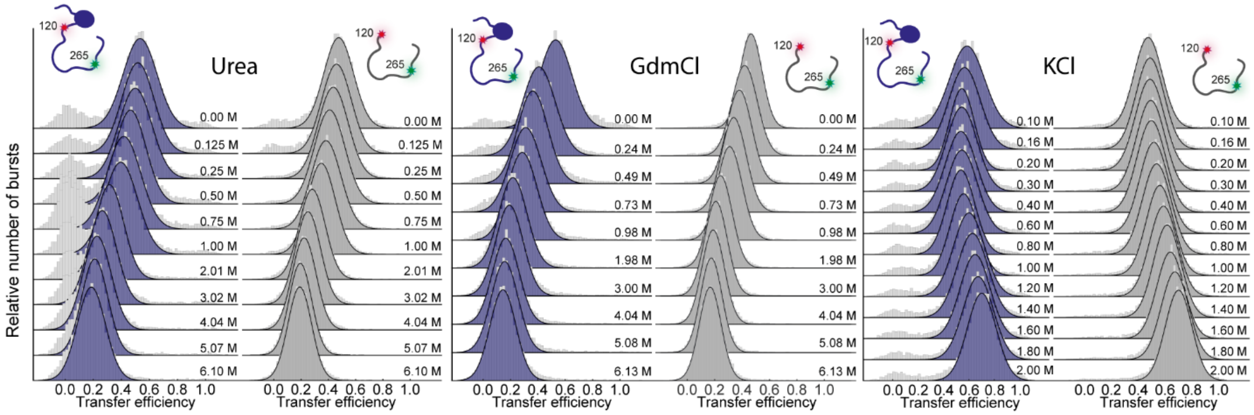

Supplementary Figure 3. Dimensions of Sox2 C-IDR change in denaturants and salt. Transfer efficiency histograms of full-length Sox2 (blue) or isolated C-IDR (grey) fluorescently labelled in positions 120-265, in different concentrations of urea (left), GdmCl (middle), and KCl (right).

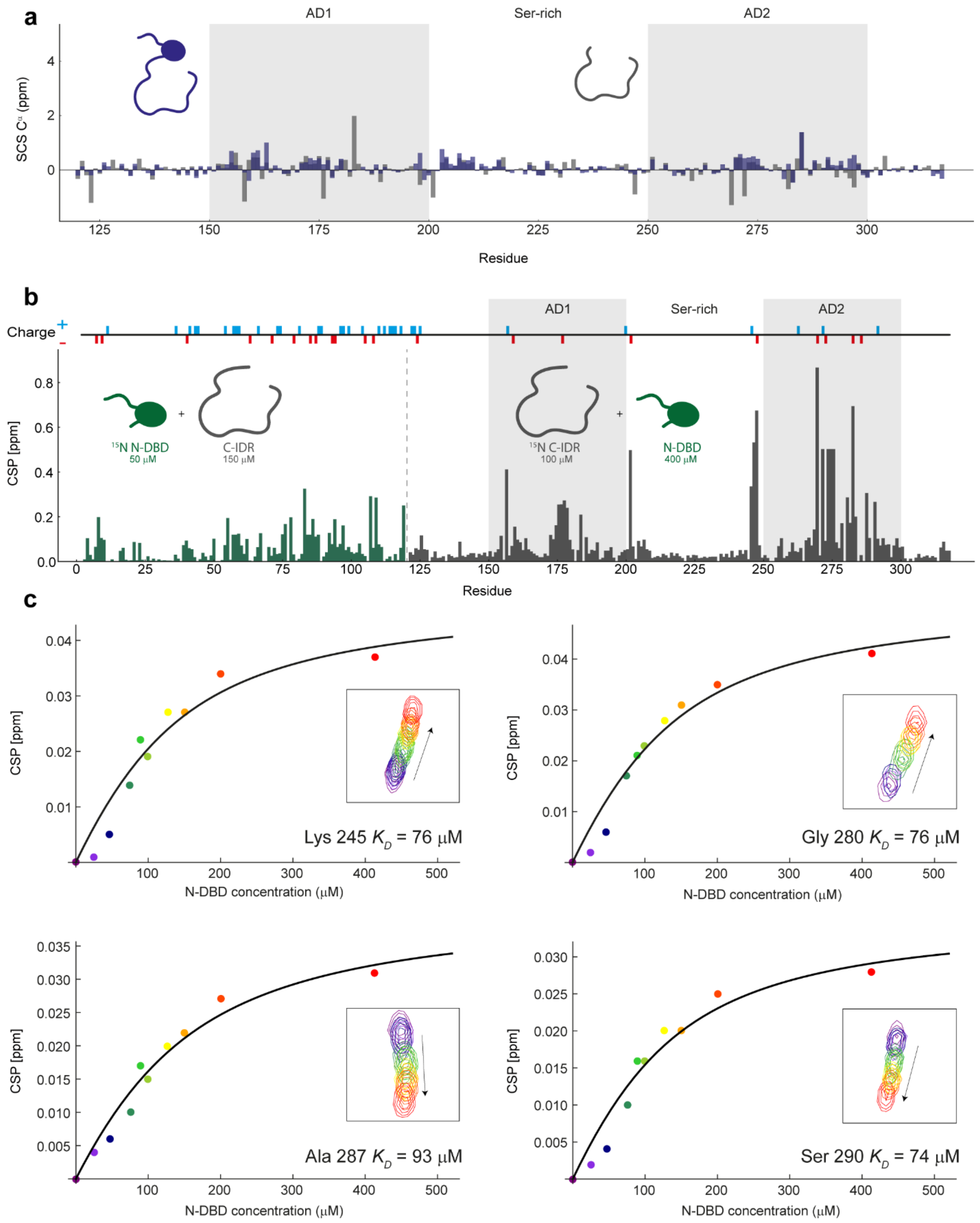

Supplementary Figure 4. NMR data for isolated domains demonstrate interdomain interactions. a) C$^{\alpha}$ SCS plot for residues 120-317 for full-length Sox2 and the isolated C-IDR. Secondary structure content is very similar in the two constructs and indicates general lack of structure. The main domains are indicated.

b) CSP plot of ^{15}N-labelled individual domains mixed with their unlabelled counterpart domain. The left side of the plot contains the combined ^{1}H,^{15}N CSPs (see Methods) for the isolated ^{15}N-labelled N-DBD (50 µM) with unlabelled C-IDR (150 µM). The right side of the plot contains the CSPs for the isolated ^{15}N-labelled C-IDR (100 µM) with unlabelled N-DBD (400 µM). **c)** Binding isotherms using chemical shifts of selected residues as a function of unlabelled N-DBD concentration. The inset panels show the corresponding resonance peaks with the colors matching the specific concentration point in the binding isotherm. Using these four residues and fitting to the simplest binding event of a 1:1 interaction, we determined an average dissocation constant K_D= 80 ± 4 µM.

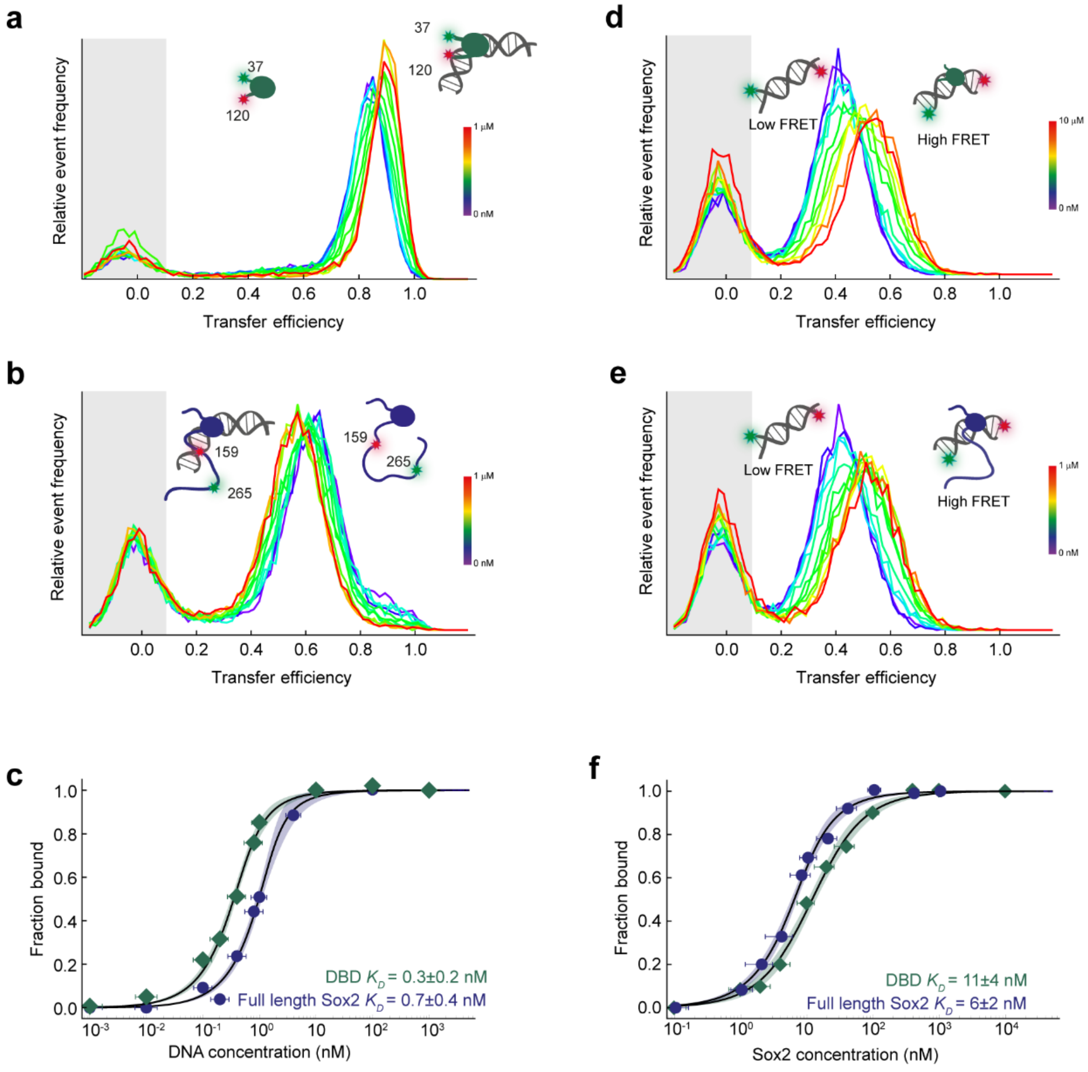

Supplementary Figure 5. Binding affinity of Sox2 to specific and non-specific DNA. a-b) Single-molecule transfer efficiency histograms of the a) isolated Sox2 DBD fluorescently labelled in positions 37 and 120 or b) full-length Sox2 fluorescently labelled in positions 159-265, with varying concentrations of unlabelled 30 bp specific DNA. **c)** The corresponding binding isotherms for panels a) and b). **d-e)** Single-molecule transfer efficiency histograms of 15 bp non-specific DNA fluorescently labelled at the 5´ and 3´-ends (see Supplementary Table 3) with varying concentrations of d) unlabelled isolated DBD or e) unlabelled full-length Sox2. **f)** The corresponding binding isotherms for panels d) and e). The dissociation constant for specific DNA using labelled proteins is nearly identical to the one determined with labelled DNA, excluding adverse effects from the fluorophores. Grey boxes in transfer efficiency histograms indicate donor-only populations, shaded areas in binding isotherms represent 95% confidence intervals of the fits, and error bars are from propagated dilution errors. All measurements were performed with 200 mM KCl.

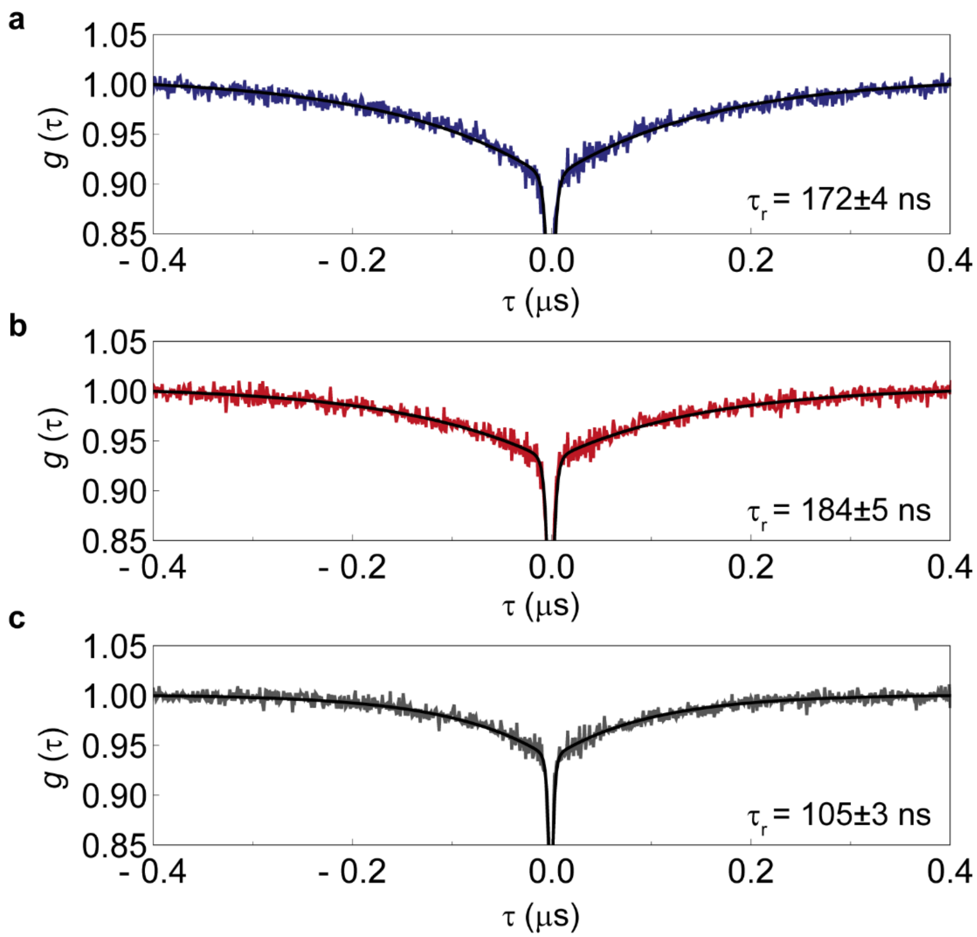

Supplementary Figure 6. Rapid reconfiguration dynamics of the Sox2 C-IDR. Nanosecond fluorescence correlation spectroscopy (nsFCS) of full-length Sox2 labelled in positions 120 and 315, and the isolated C-IDR labelled in positions 120 and 265. Fits of the donor-acceptor cross-correlation decays show sub-microsecond fluorescence intensity relaxation times for **a)** free Sox2 which persist in **b)** DNA-bound Sox2, and **c)** the isolated C-IDR. Errors are standard errors from the fits.

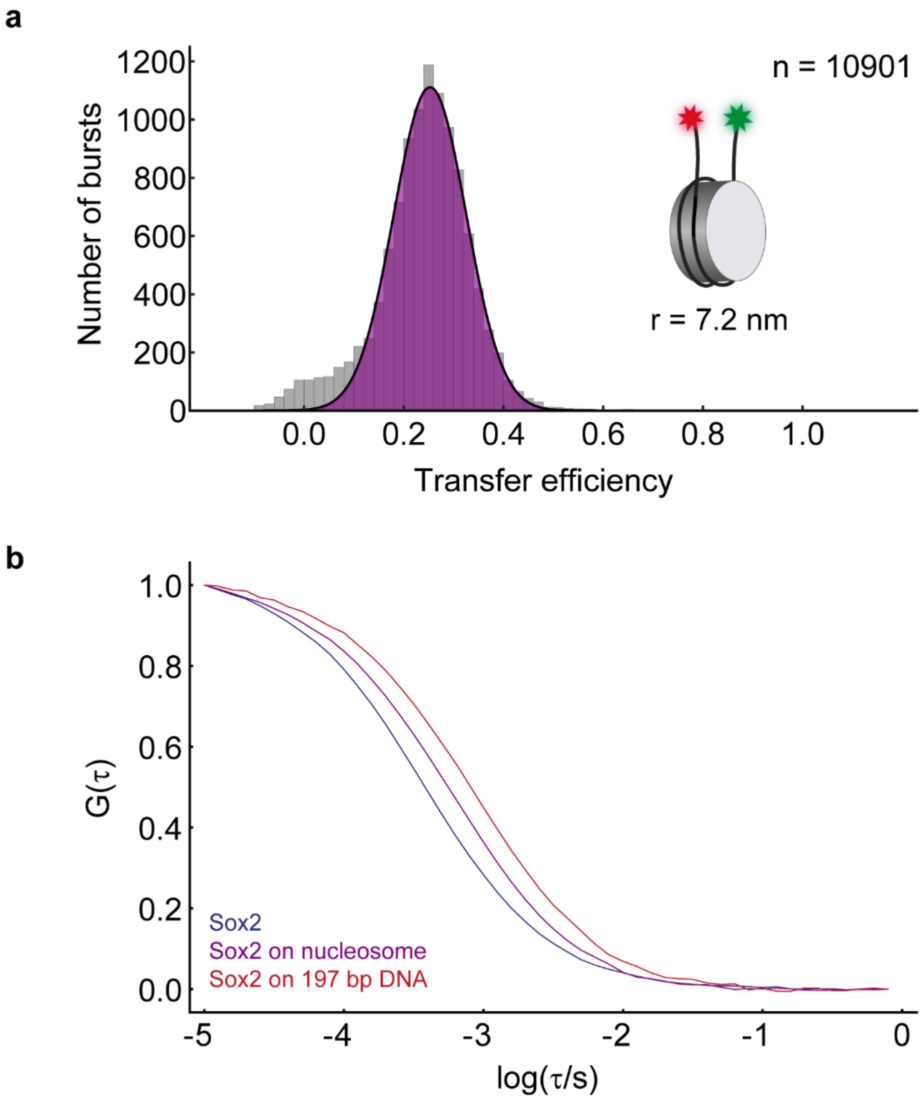

Supplementary Figure 7. Single-molecule spectroscopy analysis of nucleosomes. **a)** Single-molecule transfer efficiency histogram of 197 bp Widom 601 nucleosome fluorescently labelled at the DNA linker ends. Even at 100 pM concentrations, the nucleosome is stably wrapped as evident from the significant FRET between the fluorophores on each linker, in agreement with previous results[2]. The distance between the dyes is indicated, as calculated from the Förster equation. **b)** Donor-acceptor cross-correlation of fluorescently labelled Sox2, labelled in positions 120 and 315. Free Sox2 (blue) has a relatively short diffusion time through the confocal volume. In the presence of 90 nM of the same nucleosome as in panel a (unlabelled) the diffusion time is considerably increased (purple). In the presence of 197 bp 601 Widom DNA (red, without histone octamer), the diffusion time is increased even more, due to the much larger hydrodynamic radius of the DNA.

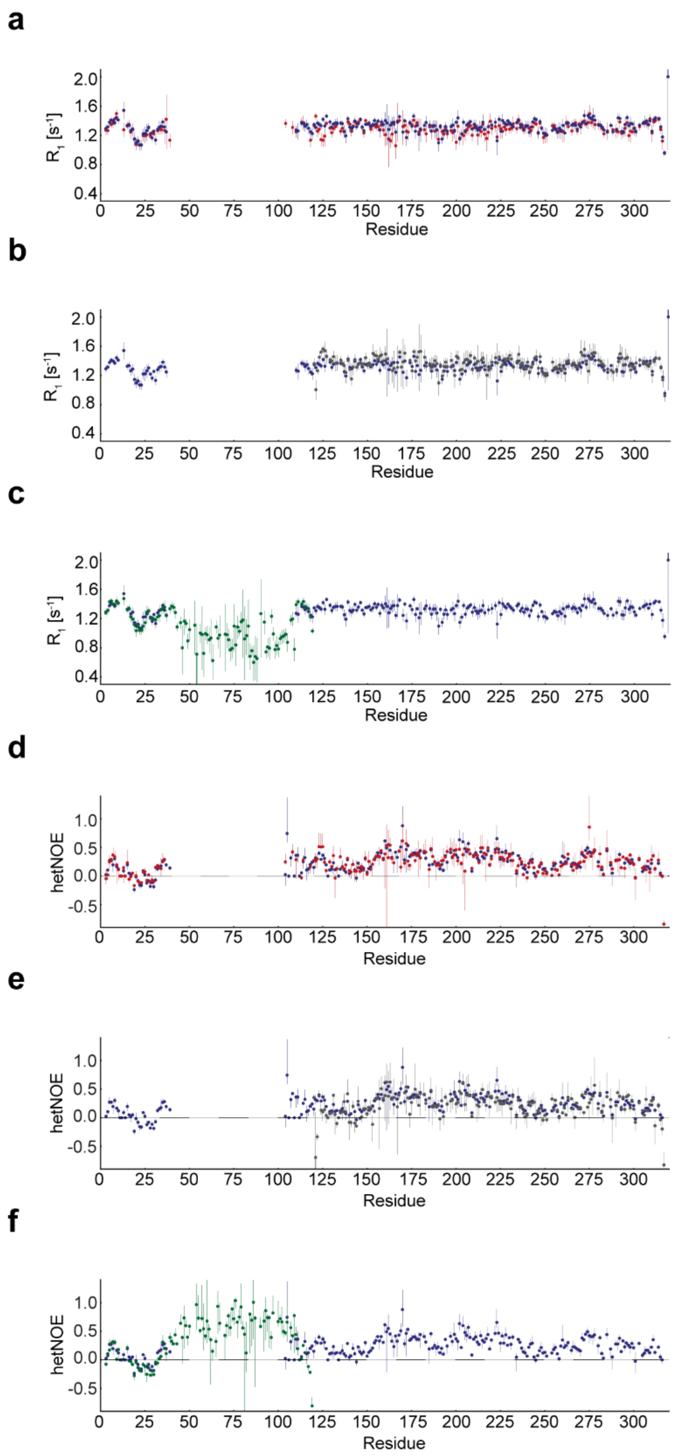

Supplementary Figure 8. Relaxation data for full-length Sox2 and the isolated C-IDR, free and DNA-bound. a-c) R_1 relaxation rates, and **d-f)** heteronuclear Overhauser effects (hetNOEs), for full-length Sox2 (blue), full-length Sox2 in complex with DNA (red), the isolated C-IDR (grey), and the isolated DBD (green). Error bars represent 95% confidence intervals from exponential fits of relaxation decays.

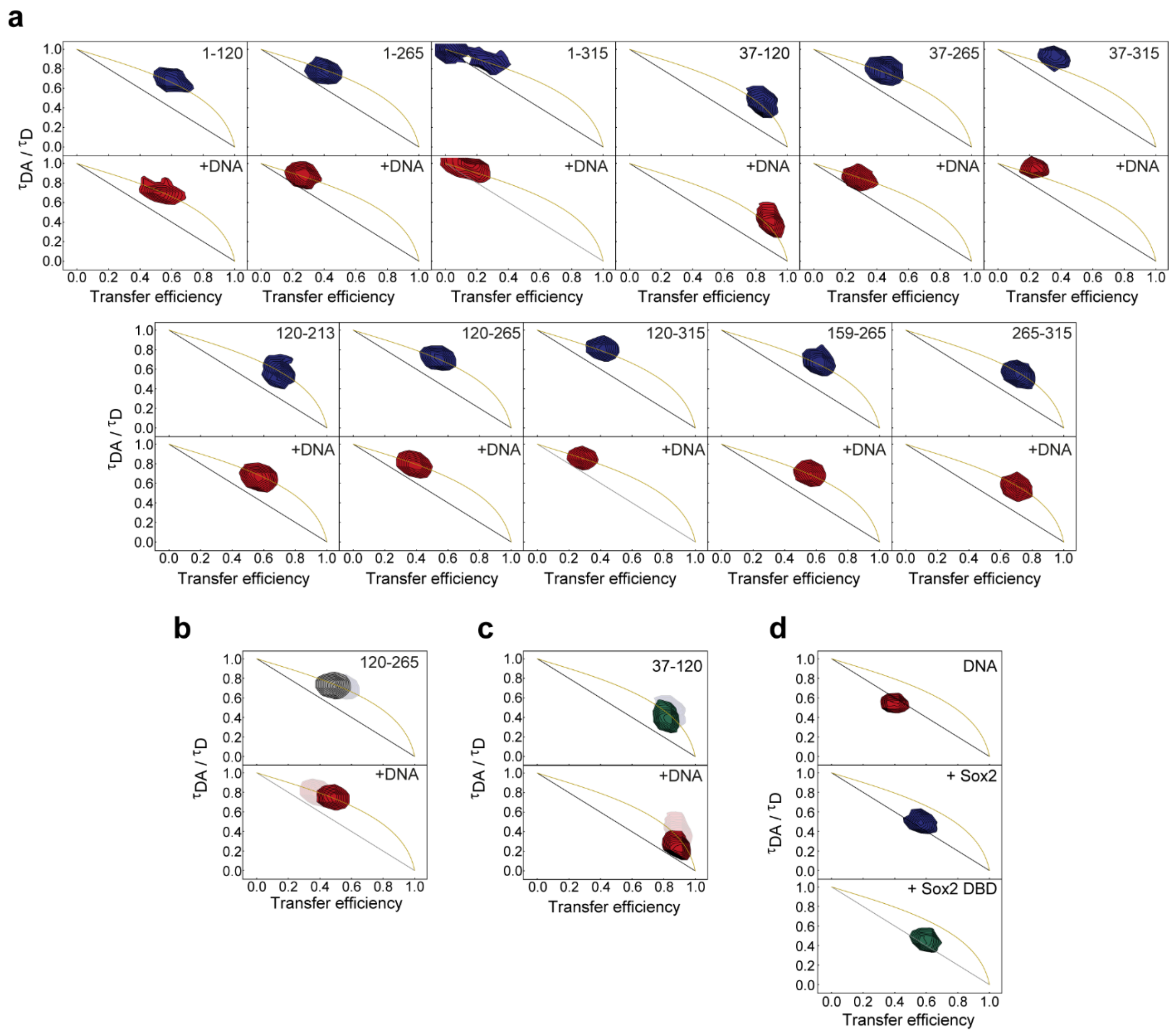

Supplementary Figure 9. Fluorescence lifetime analysis of fluorescently labelled Sox2 variants and DNA. **a)** Fluorescence lifetime analysis of all fluorescently labelled Sox2 variants, in the absence and presence of specific DNA. **b-c)** Fluorescence lifetime analysis of the isolated C-IDR (b) and DBD (c), in the absence and presence of DNA. The faint peaks represent the lifetimes of the full-length protein labelled in the same positions for comparison. **d)** Fluorescence lifetime analysis of fluorescently labelled 15 bp DNA in the free state (top), bound to full-length Sox2 (middle), and to the isolated DBD (bottom). Black lines describes the dependence of a static distance from the Förster equation, the yellow lines describe the dependence for a SAW-ν distance distribution[3].

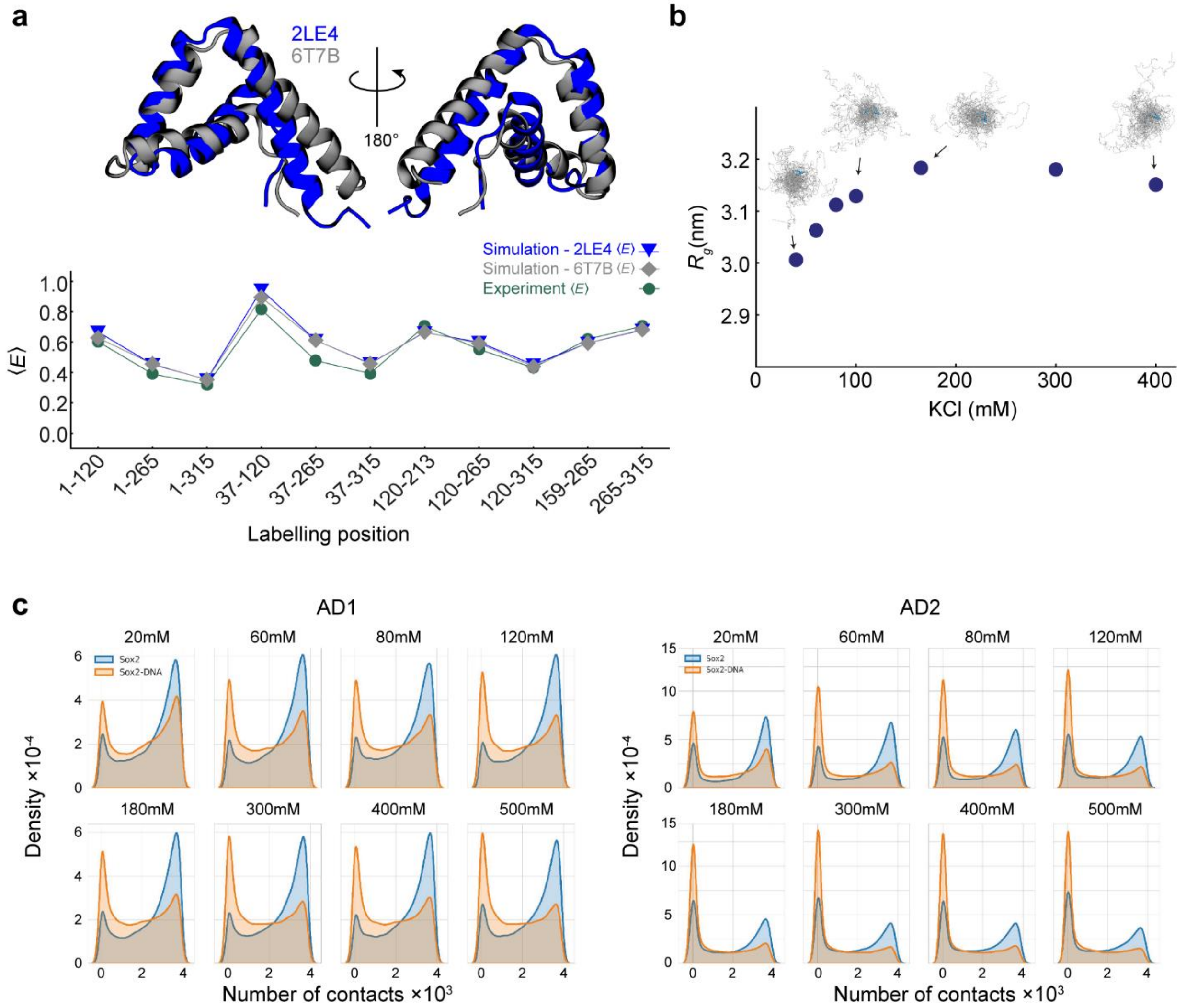

Supplementary Figure 10. Simulations under different conditions. a) Agreement between FRET efficiencies from experiments and simulations using an NMR structure of the DBD (PDB ID: 2LE4). Simulations were performed exactly as those in the main text (also shown here for PDB 6T7B). The ρ_c values of 0.89 (2LE4) and 0.92 (6T7B) show that the effects of DBD structure on the dimensions of the IDRs are minimal. An alignment of the two structures is shown (RMSD 4.4 Å). **b)** Radius of gyration, R_g, as a function of apparent salt concentration for the region encompassing residues 120-265 for free Sox2, calculated from the simulations. At low salt the chain is compact (low R_g) and then expands due to increased charge screening with increasing salt concentrations, in agreement with FRET data (Fig. 2f). 20 overlaid snapshots from the simulations at different salt concentrations are shown. **c)** Probability distributions of the contacts between AD1 and AD2 with the DBD, for Sox2 in the free and DNA-bound states, at different salt concentrations.

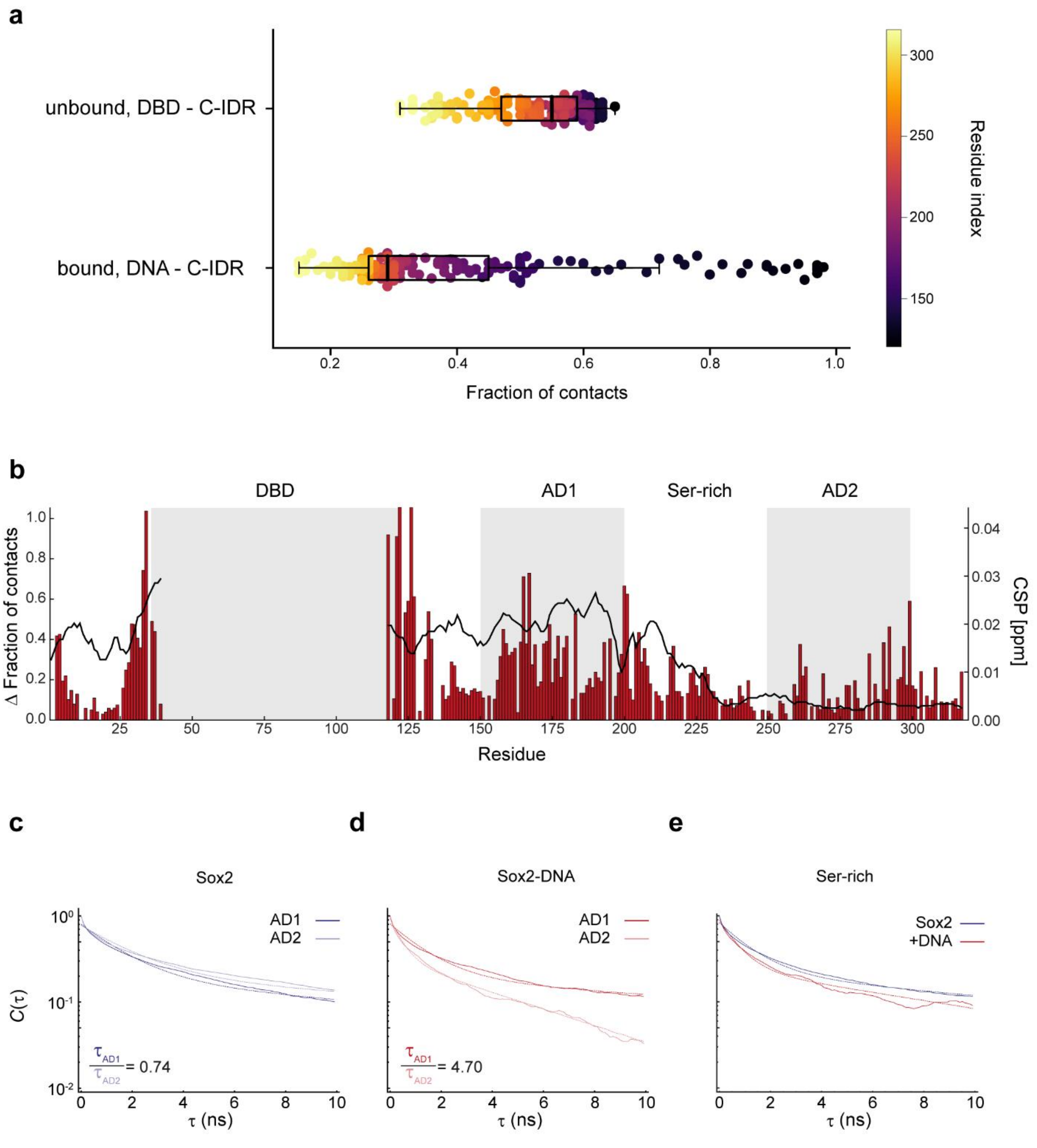

Supplementary Figure 11. Simulation analysis of inter-residue contact changes upon DNA binding. a) Fraction of contacts between the C-IDR and DBD for free Sox2, and for the C-IDR and DNA in the DNA-bound state, calculated from the simulations. The boxes represent the interquartile range and the thick lines within the boxes show the median values of the distributions. **b)** Plot showing the difference in fraction of contacts between the DBD and the IDRs (black line) from the simulations and the CSPs (red bars, from Fig. 3h) from the NMR experiments, comparing free and DNA-bound Sox2. **c-e)** Contact relaxation times between the DBD and AD1 or AD2 in the absence (**c**) and presence (**d**) of DNA, calculated

from simulations. Double-exponential fits (dotted lines) reveal that the contact relaxation times of both ADs are approximately equal in free Sox2 but diverge in complex with DNA, where contact relaxation times are more than four times as long for AD1 compared to AD2. **e)** Contact relaxation times of the Ser-rich domain, calculated from simulations. The contact relaxation times for the Ser-rich region are similar for free and DNA-bound Sox2.

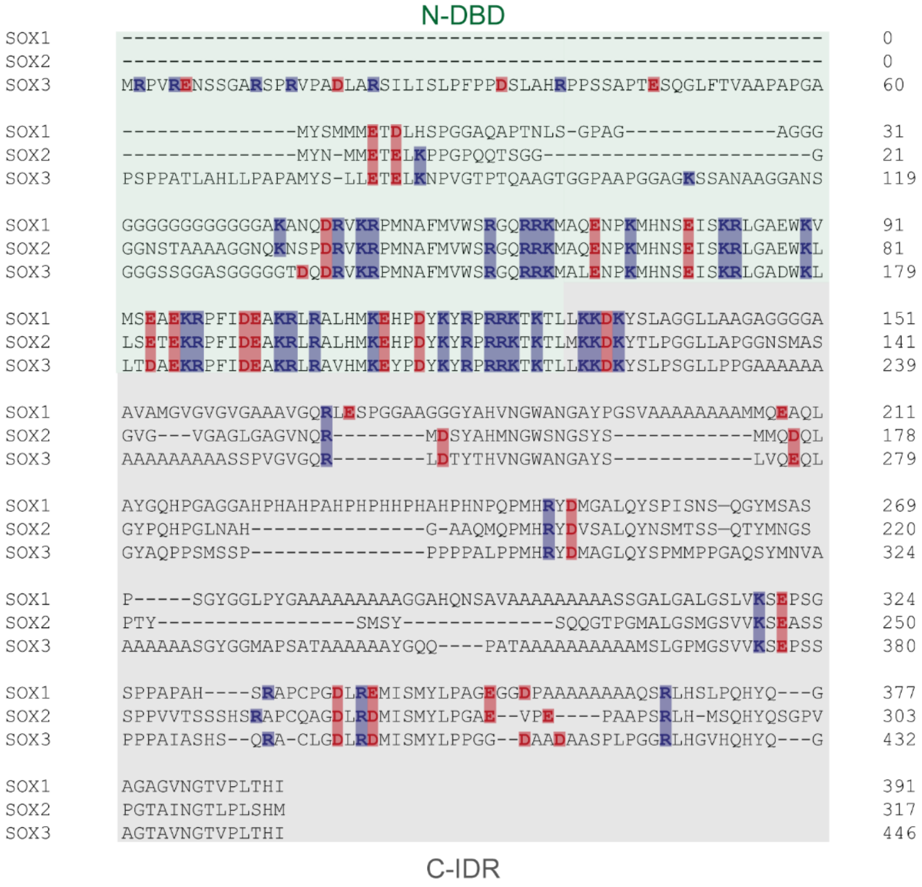

Supplementary Figure 12. Sequence alignment of the SoxB family of TFs. N-DBD and C-IDR regions are indicated with green and gray background, respectively. Charges are highlighted for clarity. Alignment generated by CLUSTAL O[4]. UniProt IDs: O00570 (Sox1), P48431 (Sox2) and P41225 (Sox3).

Supplementary Table 1. Sox2 variants that were used in this study. The residues used for fluorophore labelling (after substituting for cysteine) are marked in red.

Full-length Sox2	MYNMMETELKPPGPQQTSGGGGGNSTAAAAGGNQKNSPDRVKRPMNAFMVWSRGQRRKMAQENP<br>KMHNSEISKRLGAEWKLLSETEKRPFIDEAKRLRALHMKEHPDYKYRPRRKTKTLMKKDKYTLPGGLLAPG<br>GNSMASGVGVGAGLGAGVNQRMDSYAHMNGWSNGSYSMMQDQLGYPQHPGLNAHGAAQMQP<br>MHRYDVSALQYNSMTSSQTYMNGSPTYSMSYSQQGTPGMALGSMGSVVKSEASSSPPVVTSSSHSRAP<br>CQAGDLRDMISMYLPGAEVPEPAAPSRLHMSQHYQSGPVPGTAINGTLPLSHM
C-IDR	MKKDKYTLPGGLLAPGGNSMASGVGVGAGLGAGVNQRMDSYAHMNGWSNGSYSMMQDQLGYPQHP<br>GLNAHGAAQMQPMHRYDVSALQYNSMTSSQTYMNGSPTYSMSYSQQGTPGMALGSMGSVVKSEASSS<br>PPVVTSSSHSRAPCQAGDLRDMISMYLPGAEVPEPAAPSRLHMSQHYQSGPVPGTAINGTLPLSHM
N-DBD	MYNMMETELKPPGPQQTSGGGGGNSTAAAAGGNQKNSPDRVKRPMNAFMVWSRGQRRKMAQEN<br>PKMHNSEISKRLGAEWKLLSETEKRPFIDEAKRLRALHMKEHPDYKYRPRRKTKTL
DBD	KNSPDRVKRPMNAFMVWSRGQRRKMAQENPKMHNSEISKRLGAEWKLLSETEKRPFIDEAKRLRALH<br>MKEHPDYKYRPRRKTKTLMKK

Supplementary Table 2. Measured FRET efficiencies and fluorescence lifetimes of all protein variants and DNA. The average donor and acceptor lifetimes are 2.72±0.11 ns and 3.01±0.04 ns (n=29), respectively.

Sox2 variant	FRET efficiency (E)	Donor lifetime (ns)	Acceptor lifetime (ns)	FRET lifetime (ns)
1-120	0.60	2.80	2.95	2.92
1-120 + DNA	0.51	2.79	2.94	2.97
1-265	0.39	2.77	2.98	2.95
1-265 + DNA	0.25	2.71	2.95	2.99
1-315	0.32	2.73	3.05	3.04
1-315 + DNA	0.19	2.72	3.03	2.94
37-120	0.81	2.75	3.04	3.09
37-120 + DNA	0.88	2.72	3.01	3.03
37-265	0.48	2.71	3.04	3.07
37-265 + DNA	0.28	2.70	3.03	3.07
37-315	0.39	2.51	3.05	3.05
37-315 + DNA	0.22	2.57	3.05	3.05
120-213	0.71	2.78	3.02	2.98
120-213 + DNA	0.56	2.82	3.01	3.06
120-265	0.55	2.72	3.03	3.05
120-265 + DNA	0.37	2.72	3.01	3.04
120-315	0.43	2.62	3.04	3.03
120-315 + DNA	0.28	2.72	3.03	3.06
159-265	0.62	2.62	2.99	3.07
159-265 + DNA	0.55	2.74	2.98	3.00
265-315	0.70	2.73	3.02	3.04
265-315 + DNA	0.69	2.76	3.03	3.04
C-IDR 120-265	0.48	2.62	3.01	3.08
C-IDR 120-265 + DNA	0.48	2.61	3.00	3.00
DBD 37-120	0.81	2.46	3.00	2.98
DBD 37-120 + DNA	0.88	3.01	3.00	3.04
Labelled 15 bp DNA	0.40	2.89	3.11	3.11
Labelled 15 bp DNA + Sox2	0.59	2.81	3.03	3.04
Labelled 15 bp DNA + Sox2 DBD	0.59	2.78	2.94	3.05

Supplementary Table 3. DNA constructs for binding experiments. The Sox2 binding site in the 601-Widom sequence is marked in red. 5AmMC6 indicates a C6-amino group for labelling with an NHS-ester fluorophore.

15 bp specific, (+) strand	5'-/5AmMC6/ACT CTT TGT TTG GAT-3'
15 bp specific, (-) strand	3'-TGA GAA ACA AAC CTA/5AmMC6/-5'
15 bp nonspecific, (+) strand	5'-/5AmMC6/ TCT ATC TGT GTA TGT-3'
15 bp nonspecific, (-) strand	3'- AGA TAG ACA CAT ACA/5AmMC6/-5'
30 bp specific, (+) strand	5'-ATC CCA TTA GCA TCC AAA CAA AGA GTT TTC-3'
30 bp specific, (-) strand	3'-TAG GGT AAT CGT AGG TTT GTT TCT CAA AAG-5'
21 bp specific, (+) strand	5'-GAA TAC TCT TTG TTT GGA TGC-3'
21 bp specific, (-) strand	3'-CTT ATG AGA AAC AAA CCT ACG -5'
'601'-Widom sequence with Sox2 binding site at SHL +6, forward strand	5'-TCCATGGACCCTATACGCGGCCGCCCTGGAGAATCCCGGTGCCGAGGCCGCTCAATTGGTCG TAGACAGCTCTAGCACCGCTTAAACGCACGTACGCGCTGTCCCCCGCGTTTTAACCGCCAAG GGGATTACTCCCTAGTCTCCAGGCCTTTGTTATGCAAATACATCCTGTGCATGTATTGAACA GCAGTATGCCT-3'
'601'-Widom sequence with Sox2 binding site at SHL +6, reverse strand	3'-AGGCATACTGCTGTTCAATACATGCACAGGATGTATTTGCATAACAAAGGCCTGGAGACTAG GGAGTAATCCCCTTGGCGGTTAAAACGCGGGGGACAGCGCGTACGTGCGTTTAAGCGGTGC TAGAGCTGTCTACGACCAATTGAGCGGCCTCGGCACCGGGATTCTCCAGGGCGGCCGCGTAT AGGGTCCATGGA-5'
'601'-Widom primer, forward	5'-/5AmMC6/TCCATGGACCCTATACGCGGCCGCC-3'
'601'-Widom primer, reverse	3'-AGGCATACTGCTGTTCAATACATGCACAGGATGTATTTGCATAACAAAGGCCTGGAGAC/5AmMC6/-5'

Supplementary Table 4. Binding affinities of Sox2 for DNA from smFRET experiments, measured at 200 mM salt concentration. Errors are standard errors based on propagating pipetting errors.

**Binding reaction**	**K_D (nM)**
Labelled specific DNA – Full-length Sox2	0.4 ± 0.2
Labelled specific DNA – DBD	0.3 ± 0.1
Labelled Full-length Sox2 – 30 bp specific DNA	0.7 ± 0.4
Labelled DBD – 30 bp specific DNA	0.3 ± 0.2
Labelled non-specific DNA – Full-length Sox2	6 ± 2
Labelled non-specific DNA – DBD	11 ± 4

Supplementary Table 5. Contact relaxation times obtained from simulations for AD1, AD2, and Ser-rich domain, based on exponential fitting. Errors are the standard errors of the exponential fit.

	**τ1 (ns)**	**τ2 (ns)**
AD1	1.58 ± 0.03	18.12 ± 0.33
AD1 + DNA	1.32 ± 0.02	21.09 ± 0.27
AD2	1.95 ± 0.03	24.34 ± 0.38
AD2 + DNA	0.48 ± 0.01	4.49 ± 0.06
Ser-rich	1.20 ± 0.02	16.09 ± 0.21
Ser-rich + DNA	0.71 ± 0.01	19.17 ± 0.122

Supplementary references

1 Lescop, E., Kern, T. & Brutscher, B. Guidelines for the use of band-selective radiofrequency pulses in hetero-nuclear NMR: example of longitudinal-relaxation-enhanced BEST-type 1H-15N correlation experiments. *J. Magn. Reson.* **203**, 190-198 (2010).

2 Heidarsson, P. O. *et al.* Release of linker histone from the nucleosome driven by polyelectrolyte competition with a disordered protein. *Nat Chem* **14**, 224-231 (2022).

3 Zheng, W. *et al.* Inferring properties of disordered chains from FRET transfer efficiencies. *J. Chem. Phys.* **148**, 123329 (2018).

4 Madeira, F. *et al.* Search and sequence analysis tools services from EMBL-EBI in 2022. *Nucleic Acids Res* **50**, W276-W279 (2022).
