# Supplementary material for: DNA binding redistributes activation domain ensemble and accessibility in pioneer factor Sox2
Source: Nat Commun. 2024 Feb 16;15:1445. doi: 10.1038/s41467-024-45847-2 (PMC10873366; doi:10.1038/s41467-024-45847-2)
Supplement: Supplementary file 3 — Reporting Summary [file 41467_2024_45847_MOESM3_ESM.pdf]

nature portfolio

Corresponding author(s): Pétur O. Heidarsson

Last updated by author(s): Jan 8, 2024

Reporting Summary

Nature Portfolio wishes to improve the reproducibility of the work that we publish. This form provides structure for consistency and transparency in reporting. For further information on Nature Portfolio policies, see our Editorial Policies and the Editorial Policy Checklist.

Statistics

For all statistical analyses, confirm that the following items are present in the figure legend, table legend, main text, or Methods section.

n/a	Confirmed	
☐	☒	The exact sample size (*n*) for each experimental group/condition, given as a discrete number and unit of measurement
☐	☒	A statement on whether measurements were taken from distinct samples or whether the same sample was measured repeatedly
☒	☐	The statistical test(s) used AND whether they are one- or two-sided<br>*Only common tests should be described solely by name; describe more complex techniques in the Methods section.*
☒	☐	A description of all covariates tested
☒	☐	A description of any assumptions or corrections, such as tests of normality and adjustment for multiple comparisons
☐	☒	A full description of the statistical parameters including central tendency (e.g. means) or other basic estimates (e.g. regression coefficient) AND variation (e.g. standard deviation) or associated estimates of uncertainty (e.g. confidence intervals)
☒	☐	For null hypothesis testing, the test statistic (e.g. *F*, *t*, *r*) with confidence intervals, effect sizes, degrees of freedom and *P* value noted<br>*Give P values as exact values whenever suitable.*
☒	☐	For Bayesian analysis, information on the choice of priors and Markov chain Monte Carlo settings
☒	☐	For hierarchical and complex designs, identification of the appropriate level for tests and full reporting of outcomes
☒	☐	Estimates of effect sizes (e.g. Cohen's *d*, Pearson's *r*), indicating how they were calculated

Our web collection on statistics for biologists contains articles on many of the points above.

Software and code

Policy information about availability of computer code

Data collection	All data requiring software was collected using PicoQuant SymphoTime 64; modified Gromacs 4.5.4, TopSpin (Bruker)
Data analysis	A custom module for Mathematica v12.3 (Wolfram Research) was used for the analysis of single-molecule fluorescence data. It is available at https://schuler.bioc.uzh.ch/programs/. NMR data was processed and analyzed using NMRPipe 11.0, TopSpin 4.0.7 and CCPN Analysis 2.5.2. For simulations MDAnalysis 2.60, in-house scripts, USCF Chimera 1.17.3 and VMD 1.9.3 were used to analyse the trajectories.

For manuscripts utilizing custom algorithms or software that are central to the research but not yet described in published literature, software must be made available to editors and reviewers. We strongly encourage code deposition in a community repository (e.g. GitHub). See the Nature Portfolio guidelines for submitting code & software for further information.

Data

Policy information about availability of data

All manuscripts must include a data availability statement. This statement should provide the following information, where applicable:

- Accession codes, unique identifiers, or web links for publicly available datasets
- A description of any restrictions on data availability
- For clinical datasets or third party data, please ensure that the statement adheres to our policy

Data supporting the findings of this paper are available from the corresponding authors upon request. The simulation trajectories have been deposited to the

Protein Ensemble Database (PED00439 and PED00440). Chemical shift assignments were deposited to the Biological Magnetic Resonance Data Bank (BMRB accession number 51964). Source data are provided with this paper.

Research involving human participants, their data, or biological material

Policy information about studies with human participants or human data. See also policy information about sex, gender (identity/presentation), and sexual orientation and race, ethnicity and racism.

Reporting on sex and gender	N/A
Reporting on race, ethnicity, or other socially relevant groupings	N/A
Population characteristics	N/A
Recruitment	N/A
Ethics oversight	N/A

Note that full information on the approval of the study protocol must also be provided in the manuscript.

Field-specific reporting

Please select the one below that is the best fit for your research. If you are not sure, read the appropriate sections before making your selection.

☒ Life sciences ☐ Behavioural & social sciences ☐ Ecological, evolutionary & environmental sciences

For a reference copy of the document with all sections, see nature.com/documents/nr-reporting-summary-flat.pdf

Life sciences study design

All studies must disclose on these points even when the disclosure is negative.

Sample size	The sample size was chosen according to standard throughput of the corresponding experiment and/or instrument. At least three independent replicates were performed for statistical analysis. In single-molecule FRET experiments, each replicate represents > 5000 individual molecules.
Data exclusions	Bursts arising from molecules with only a single fluorophore and with less than 50 photons were excluded from the analysis.
Replication	The consistency of the experimental outcomes was verified through repeated trials on freshly prepared samples under varied experimental parameters, in at least three independent trials. This process was conducted to ascertain the degree of variation in the observed results in response to changes in the experimental conditions.
Randomization	This study did not allocate experimental groups and randomization was therefore not required.
Blinding	Experiments were not blinded as none included subjective human assessment. Furthermore, all samples were prepared, measured and analysed under the same stringent conditions.

Reporting for specific materials, systems and methods

We require information from authors about some types of materials, experimental systems and methods used in many studies. Here, indicate whether each material, system or method listed is relevant to your study. If you are not sure if a list item applies to your research, read the appropriate section before selecting a response.

Materials & experimental systems

n/a	Involved in the study	
☒	☐	Antibodies
☒	☐	Eukaryotic cell lines
☒	☐	Palaeontology and archaeology
☒	☐	Animals and other organisms
☒	☐	Clinical data
☒	☐	Dual use research of concern
☒	☐	Plants

Methods

n/a	Involved in the study	
☒	☐	ChIP-seq
☒	☐	Flow cytometry
☒	☐	MRI-based neuroimaging

Plants

Seed stocks	N/A
Novel plant genotypes	N/A
Authentication	N/A
