# Supplementary material for: DNA binding redistributes activation domain ensemble and accessibility in pioneer factor Sox2
Source: Nat Commun. 2024 Feb 16;15:1445. doi: 10.1038/s41467-024-45847-2 (PMC10873366; doi:10.1038/s41467-024-45847-2)
Supplement: Supplementary file 4 — Description of Additional Supplementary Files [file 41467_2024_45847_MOESM4_ESM.pdf]

Description of Additional Supplementary Files

Supplementary Movie Legends

Supplementary Movie 1: Simulation of free Sox2. The free Sox2 ensemble, with the Sox2 DBD shown in blue and the disordered N-IDR and CIDR in silver. The movie shows 400 conformations of the Sox2 ensemble, collected from the 16 µs Langevin dynamics simulations (see Methods).

Supplementary Movie 2: Simulation of Sox2 in complex with DNA. Ensemble of Sox2 bound to DNA, with the Sox2 DBD shown in blue, the disordered N-IDR and CIDR in silver, and the DNA in dark grey. The movie shows 400 conformations of the Sox2 ensemble in complex with DNA, collected from the 16 µs Langevin dynamics simulations (see Methods).
